# Responding to chemical weapons violations in Syria: legal, health, and humanitarian recommendations

**DOI:** 10.1186/s13031-018-0143-3

**Published:** 2018-02-19

**Authors:** Julia Brooks, Timothy B. Erickson, Stephanie Kayden, Raul Ruiz, Stephen Wilkinson, Frederick M. Burkle

**Affiliations:** 1 Harvard Humanitarian Initiative, Cambridge, MA USA; 2 Department of Emergency Medicine, Brigham and Women’s Hospital; Harvard Medical School, Harvard Humanitarian Initiative, Cambridge, MA USA; 3 Department of Emergency Medicine, Brigham and Women’s Hospital, Harvard Medical School; and Core Faculty, Harvard Humanitarian Initiative, Cambridge, MA USA; 4 U.S. House of Representatives for California’s 36th congressional district, Washington, D.C. USA

**Keywords:** Syria, Chemical weapons, Nerve agents, Disaster preparedness, Conflict zones, Humanitarian response, Protection of civilians, International law, International treaties, United Nations

## Abstract

**Background:**

The repeated use of prohibited chemical weapons in the Syrian conflict poses serious health, humanitarian, and security threats to civilians, healthcare personnel, and first responders. Moreover, the use of chemical weapons constitutes a clear and egregious violation of international law—likely amounting to a war crime—for which continued impunity is setting a dangerous precedent in relation to current and future conflicts. This debate article calls upon concerned states, organizations, and individuals to respond urgently and unequivocally to this serious breach of international legal and humanitarian norms.

**Main Body:**

Based on health, humanitarian, and legal findings, this article calls for concrete action to: 1) reduce the risk of chemical weapons being used in current and future conflicts; 2) review and support the preparedness equipment and antidote supplies of first responders, humanitarian organizations, and military forces operating in Syria; 3) support international mechanisms for monitoring and enforcing the prohibition on chemical weapons, including through criminal accountability; 4) support civilian victims of chemical weapons attacks, including refugees; and 5) re-commit to the complete elimination of chemical weapons in compliance with the *Chemical Weapons Convention* (1993), a comprehensive treaty that bans chemical weapons and requires their complete destruction.

**Conclusion:**

All involved states and organizations should take urgent steps to ensure the protection of the most vulnerable victims of conflict, including victims of chemical weapons attacks in Syria, and to reinforce international law in the face of such serious violations.

## Background

In the deadliest use of chemical weapons in Syria since August 2013, at least 83 people were killed, including 28 children, and over 293 people were reported injured by a confirmed sarin gas attack on the northern rebel-held area of Khan Shaykhun, Idlib Province, Syria on April 4, 2017, carried out by the Syrian government [1]. The attack followed in a pattern of repeated use of chemical weapons in the Syrian conflict which poses serious health, humanitarian, and security threats to civilians, healthcare personnel, and first responders in Syria. Moreover, the use of chemical weapons constitutes a clear and egregious violation of international law—in particular, the 1993 Chemical Weapons Convention (CWC)—likely amounting to a war crime for which continued impunity is already setting a dangerous precedent for current and future conflicts and imperiling global public health.

## History of chemical warfare in Syria and elsewhere

The use of chemical weapons is rare in modern warfare. The first modern use of large-scale chemical warfare dates back to World War I, when all major belligerents used or attempted to use chlorine gas, mustard agents, and/or phosgene, killing an estimated 100,000 troops. In 1988, Iraqi leader Saddam Hussein used mustard gas and nerve agents against Iranian ground forces and Kurdish rebels, killing thousands [2].

The alleged use of internationally banned chemical weapons such as sarin and chlorine gas in Syria can be traced back to 2013, when the first reports became known in many locations, including Khan Al Asal, [3] Sarqib, [4] Ghouta, [5] and Jobar [6]. According to the most recent statistics, there have been 234 separate and documented chemical attacks since the beginning of the Syrian war, resulting in over 13,000 injuries and 3415 deaths [7]. Two hundred eleven of these attacks were attributed to chlorine gas alone, or chlorine with traces of sarin.

The United Nations (UN), on numerous occasions, has confirmed the use of chemical weapons in Syria. Between March 2013 and March 2017, the UN Human Rights Council-mandated Commission of Inquiry on Syria “documented 25 incidents of chemical weapons use in the Syrian Arab Republic, of which 20 were perpetrated by government forces and used primarily against civilians,” not including the Khan Shaykhun attack in April 2017 [8]. These incidents have entailed the use of sarin, which has been used in multiple incidents, [9] as well as other chemical weapons, foremost among them chlorine gas, which has reportedly become “almost routine” in northern Syria [10].

## Health and medical impacts of sarin gas and other nerve agents

The health and medical impacts of chemical weapons are severe, immediate, and life threatening, causing horrendous injuries and rapid death, especially for children. Upon exposure to nerve agents, for example, victims are likely to experience drooling, vomiting, and diarrhea, followed by paralysis and asphyxiation. Those who do not die are likely to suffer from long-term neurological damage. Contributing to the cruelty of these weapons, chemical attacks are particularly deadly for civilians sheltering below ground from conventional weapons attacks, since gas agents are often denser than air and can therefore transform basements or bomb shelters into death traps [11].

Exposure and poisoning by a nerve agent like sarin or venomous agent X, also known as VX, disrupts cholinergic transmission of the nerve signals throughout the body, leading to symptoms that may include constriction of pupils (miosis), profuse salivation, involuntary urination and defecation, respiratory distress, muscle paralysis, loss of consciousness, and seizures. Death may result by asphyxiation and bronchospasm due to a loss of control of the respiratory muscles and inability to clear pulmonary secretions (bronchorrhea).

Symptoms will appear within a few seconds after exposure to the vapor form, and from within a few minutes to up to several hours after exposure to the liquid form. Any liquid contact with the skin, unless washed off immediately, could be fatal. Children are more vulnerable than adults to the lethal effects because of their closer proximity to the ground, smaller body mass, higher respiratory rate, increased skin permeability, and immature metabolic systems [12].

### Response and treatment

Recovery from nerve agent exposure is possible with focused and immediate treatment, but available antidotes must be used rapidly to be effective. If victims have been exposed to a nerve agent, they should be removed from the source of the exposure and evacuated into fresh air. Skin decontamination of toxic chemical warfare nerve agents is crucial for mitigating the systemic toxicity. Contaminated clothing should be removed by rescuers wearing personal protective equipment. Rescuers should also rapidly wash and decontaminate the victims with soap and copious amounts of water and should remove and dispose of clothing in a sealed, secure biological plastic bag to avoid secondary exposure. Family members and rescue personnel risk cross-contamination and secondary toxicity if not adequately protected with latex gloves and proper equipment when handling and treating victims.

The basis for the medical management of nerve agent-poisoned casualties is derived from clinical experience with pesticide poisoning [13]. The two pillars of treatment include parenteral administration of atropine (2-6 mg every 5–10 min) to counter the muscarinic effects of excess acetylcholine, and 1-2 g of pralidoxime (2-PAM) to cleave the nerve agent from acetylcholinesterase and restore the active site. Atropine should be administered until symptoms of bradycardia, bronchospasm, and bronchorrhea resolve, a process that may require extraordinarily high doses of atropine. Current military field treatments for nerve agent intoxication include auto-injectors containing atropine and 2-PAM, which help to restore the transmission of nerve signals in the body [14]. With severe cases of nerve agent poisoning, large doses of atropine may be required, often exceeding available supplies. In many low- or middle-income countries such as Syria, 2-PAM is too costly and not readily available. A flood of victims after a nerve agent attack such as sarin gas may quickly deplete hospital supplies of atropine and 2-PAM. Moreover, although much of the current atropine supply in Syria is expired, healthcare providers should be aware that, if necessary, these antidotes may be used on an emergency basis beyond their expiration date [15].

Several leading concerns with regard to the immediate humanitarian response include:

- The absence of available technology for identifying which chemical agent has been used, impacting upon immediate response, attribution, and accountability efforts [16]
- The lack of sufficient personal protective equipment for first responders (whether civil or military)
- Insufficient antidote reserves (particularly oxides [2-PAM])
- The danger of rescuers or grieving families subjecting themselves to toxic exposure, whether from the victim’s secretions (lung secretions, vomit, diarrhea) or directly from the nerve agent itself if the skin is not properly decontaminated

Moreover, beyond the particular concerns outlined above, the ability of healthcare providers to respond to the use of chemical weapons has been severely hampered by the direct and repeated targeting of medical facilities and personnel—a war crime under international law—including by the use of conventional and chemical weapons [8]. This “weaponisation of health care” has made Syria “the most dangerous place on earth for health-care providers”: over 800 health workers have been killed in the conflict, [17] and many more injured, incarcerated, or tortured, sparking a mass exodus of healthcare workers from the country [18]. This unprecedented challenge to medical humanitarianism, and the failure of the international community to effectively respond, has given rise to growing calls for rethinking the provision of healthcare and humanitarian assistance in conflict [19].

## Strict prohibition of chemical weapons under international law

The use of chemical weapons in armed conflict not only poses a serious risk to the health of civilians but also is strictly and unambiguously prohibited under international law. Warring parties are prohibited from using chemical weapons in any situation or circumstances (in international or non-international armed conflict) against any persons (civilians or soldiers). Chemical weapons may also not be used in retaliation for a previous chemical weapons attack.

These prohibitions date back to the late 19th century [20] and were most recently articulated and developed in the 1993 CWC, which entered into force in 1997. The CWC prohibits the use, development, production, stockpiling, and transfer of chemical weapons, and it established the Organisation for the Prohibition of Chemical Weapons (OPCW) for the purposes of implementation [21]. The CWC obliges parties to: destroy all existing chemical weapons and production facilities under international verification; monitor the chemical industry to prevent the emergence of new weapons; provide assistance and protection to States Parties against chemical threats; and foster the peaceful use of chemistry. Considering its near universal membership (with 192 States Parties at present) and intrusive verification mechanisms, the CWC is widely considered to be one of the most successful disarmament treaties. Only four states—Egypt, North Korea, South Sudan, and Israel (which has signed but not ratified)—remain outside of the convention [11].

As of October 2016, the OPCW verified that approximately 90% of the world’s declared stockpile of 72,304 metric tons of chemical agent had been destroyed, [22] including 90% of the U.S.’s declared stockpile of 33,600 metric tons of chemical agent, in line with States Parties’ obligations to work towards complete disarmament.

### Syrian chemical weapons disarmament

While not previously a member, Syria agreed to join the CWC following international pressure sparked by the August 2013 Ghouta attack [23]. It became a party to the CWC in September 2013 (with entry into force in October) and reported an inventory of 1300 tons of chemical agents and precursors to the OPCW. By June 2014, the OPCW had verified the destruction of 24 of Syria’s 27 declared production and storage facilities (the remaining sites were deemed too dangerous to visit) and the removal from the country of all of Syria’s declared chemical weapons.

Since then, however, suspicions of undeclared stockpiles (including of sarin, chlorine, and ricin) and reports of chemical weapons use in Syria have persisted. UN investigators found “compelling confirmation” of the use of chemical weapons in Syria in 2014 and 2015, leading the UN to establish the “OPCW-UN Joint Investigative Mechanism,” which later attributed responsibility to the Syrian government (on three occasions) and ISIS (on one occasion) [24].

In light of the evidence of continued possession and use of chemical weapons in Syria, and the volatility and complexity of the current conflict, concerns also exist over the potential for the Syrian regime to transfer such weapons to non-state actors (such as Hezbollah in Lebanon) or to other states (such as Iran or North Korea). The risk also exists, especially in the event of regime collapse, that the Syrian regime could lose control of these weapons, resulting in further proliferation. Given the events that this section has described, it is apparent that there is an immediate need to understand the extent of remaining undeclared weapons stockpiles in Syria—including potential bio-weapons capabilities—and to work toward their destruction.

## Enforcement

As the conflict in Syria becomes increasingly complex due to a range of factors—including the conflict’s protracted nature, the plethora of fragmented armed groups operating in the country, and the geopolitical importance of the conflict—it is critical to consider the international diplomatic pressure points that may prevent Syria from using chemical weapons in the future and to hold those responsible to account. Both sanctions and criminal accountability measures are available to states and the international community to enforce the international ban on the use of chemical weapons. From a humanitarian perspective, it is also crucial that any such actions be accompanied by measures to mitigate the impact on already vulnerable populations.

### Condemnation

First, states must condemn the use of chemical weapons in the strongest possible terms. Many political leaders and international bodies have already voiced strong ethical and legal objections condemning the use of chemical weapons. However, the Security Council (SC) has failed to formally condemn the attacks or take further action to stop them. On April 12, 2017, the SC tabled a draft resolution—backed by the U.S., U.K., and France but opposed by Russia with its veto power—which would have condemned the chemical weapons attack in Khan Shaykhun and called for accountability. While continuing to work to break the gridlock in the SC, other states should individually and collectively act to forcefully condemn the use of chemical weapons in Syria.

### OPCW enforcement powers

Second, if the OPCW finds a state party to be in violation of the CWC, it may authorize sanctions. The OPCW may not authorize the use of military force, and although it may consult with the SC to do so if appropriate, the SC itself is limited both by individual veto power and the lack of a military response capability apart from Member States. Given the clear evidence of repeated violation in Syria – despite the OPCW disarmament mission – the OPCW should use its power to urge its Member States who have not already done so to institute sanctions against Syria over the use of chemical weapons.

### Sanctions

Third, sanctions constitute another available and non-military means for states to enforce the ban on chemical weapons vis-à-vis the Syrian regime. In February 2017, Russia cast its seventh SC veto (and China its sixth veto) blocking an attempt by the U.S., U.K., and France to impose collective sanctions on Syria over the use of chemical weapons. In contrast, the SC has passed sanctions against ISIS, Al-Nusra Front, Al-Qaida, and other designated terrorist organizations operating in Syria. A number of states have nonetheless applied a range of sanctions against the Syrian government and key individuals and organizations supporting the Assad regime since the beginning of the conflict, as have the regional bodies such as the European Union, the Arab League, and the Organization of Islamic Cooperation. States that have not already done so should consider instituting further sanctions against Syria for chemical weapons-related violations. However, this should be done with caution, as sanctions may also cause disproportionate harm to civilians, raising significant humanitarian concerns, as discussed in greater detail below.

### The responsibility to protect

The widespread and systematic nature of international law violations in the Syrian conflict has also sparked significant debate over the potential application of the doctrine of Responsibility to Protect (R2P). In 2005, recognizing the failure to adequately respond to the most heinous crimes known to humankind, world leaders made a commitment to protect populations from genocide, war crimes, ethnic cleansing, and crimes against humanity at the UN World Summit. This commitment under R2P stipulates that individual states carry the primary responsibility for the protection of populations from mass atrocity crimes and that the international community has a responsibility to assist states in fulfilling this responsibility. Accordingly, under R2P, the international community should use appropriate diplomatic, humanitarian, and other peaceful means to protect populations from these crimes. If a State fails to protect its populations or is in fact the perpetrator of crimes, the international community must be prepared to take stronger measures, including the collective use of force through the SC. Much of the doctrine of R2P is a restatement of existing commitments, rather than new international law, though the Syrian context merits considerable reflection on R2P and how the international community can collectively renew and live up to its commitments to civilian protection [25]. Thus far, the Russian veto on the SC has prevented a collective decision to authorize the use of force to protect civilians in Syria.

### Health & humanitarian consequences

From a humanitarian perspective, it is crucial that any enforcement measures—such as sanctions or the use of UN peace enforcement action—consider and mitigate the impact on already vulnerable civilian populations. For instance, any military action, if deemed appropriate, must be carried out in accordance with international humanitarian law (IHL), including the obligation to protect civilians from adverse impacts of the conflict [26]. Similarly, any sanction measures should ideally be multilateral, targeted at the members and supporters of the regime, accompanied by humanitarian assistance for vulnerable populations, and combined with other diplomatic efforts and incentives.

Finally, it should be noted that while the use of chemical weapons constitutes an egregious violation of international law with devastating impacts on civilians, it is far from the only such violation to be committed in the Syrian conflict which needs to be addressed. Parties to the conflict, especially the Syrian government, have routinely used other internationally sanctioned weapons, including cluster munitions and incendiary weapons, and have carried out deliberate and systematic attacks on civilians, hospitals, cultural heritage sites, and other protected persons and objects, in direct violation of international law and medical neutrality [27].

## Accountability

It is also critical to pursue individual criminal accountability for the use of chemical weapons and other serious violations of international law committed in Syria. While many of these options are currently being pursued in tandem, each faces significant barriers necessitating significantly more international support to achieve even partial justice.

### Independent Mechanism to prepare future prosecutions

In December 2016, the UN General Assembly established an “Independent Mechanism” to assist in investigation and prosecution of serious international crimes committed in Syria. This Mechanism’s aim is to supplement the work of the existing UN Commission of Inquiry on Syria by taking on a pre-prosecutorial function: consolidating, preserving, and analyzing evidence in order to prepare files to assist in future investigations and prosecutions of those individually responsible for serious international crimes. The effective operation of the Independent Mechanism will depend on sufficient funding and political support by Member States, and ultimately, the opening up of avenues for prosecution.

### International Criminal Court (ICC)

The International Criminal Court (ICC) offers one potential avenue for pursuing individual criminal accountability for the use of chemical weapons in Syria, though political and jurisdictional issues pose significant barriers to its involvement. Established by the Rome Statute in 1998 and beginning operations in 2003, the ICC is the first permanent international criminal court for the investigation and prosecution of perpetrators of the most serious international crimes, including the use of chemical weapons. Since Syria is not a State Party to the Rome Statute (and neither are Russia, the U.S., Iran, or other key parties to the Syria conflict), the ICC does not have a clear basis for jurisdiction. The ICC would be able to exercise jurisdiction over the situation in Syria if: 1) the Syrian government ratifies the Rome Statute; 2) the Syrian government accepts the jurisdiction of the ICC through a declaration; or 3) the SC refers the situation in Syria to the ICC. With regard to a referral by the SC of the situation in Syria to the ICC, this option has thus far been attempted but blocked at the SC by the vetoes of permanent members Russia and China. The ICC could, however, exercise jurisdiction over crimes committed by dual nationals of Syria and a state party to the ICC (e.g., ISIS foreign fighters who are nationals of European ICC Member States). Furthermore, if the ICC were to gain jurisdiction over the situation in Syria, the Court would still face a number of hurdles in prosecuting cases, including gaining custody of high-level accused, such as President Assad, and protecting witness and victim participation. The prosecutor would, however, benefit from the existence of a “staggering” body of evidence that has already been gathered and analyzed [28].

### Establishment of an ad hoc tribunal

Beyond the ICC, the international community—either through a SC decision or agreement with the affected state—may also set up an ad hoc international or hybrid international-domestic criminal tribunal to prosecute serious international crimes, such as the use of chemical weapons, as states have done for cases in the former Yugoslavia, Rwanda, Sierra Leone, Lebanon, and Cambodia. Some have advocated for the establishment of such an ad hoc tribunal for Syria, though the permanent ICC is generally considered to be a more viable option, given the political difficulties and costs of establishing a separate court de novo.

### Domestic prosecutions of international crimes

In addition to international criminal courts, states may also prosecute the use of prohibited chemical weapons in their domestic courts, provided that they have incorporated these international crimes into their domestic criminal codes and are able to gain custody over the accused (e.g., as refugees, asylum seekers, or foreign fighters). Several prosecutions of accused Syrian war criminals have already been initiated in European states (including Germany, France, the Netherlands, Norway, Spain, Sweden, and Switzerland) through the exercise of domestic or universal jurisdiction. These developments indicate that it may also be possible to prosecute further Syrian perpetrators who have fled to Turkey, Jordan, Lebanon, or the U.S., as well as dual Syrian nationals or foreign fighters. Given the lack of political will for accountability at the international level, these domestic cases are critically important to advancing justice in the near term. However, their overall impact on the situation in Syria is likely to be limited. In light of this reality, concerned states and organizations should support legal and investigative efforts in national jurisdictions, while at the same time continuing to investigate and prepare cases for future international prosecution and continuing diplomatic efforts to open a path to such eventual international criminal prosecution.

## Conclusion and recommendations

In light of aforementioned challenges posed by the repeated use of prohibited chemical weapons in the Syrian conflict, this article calls upon concerned states, organizations, and individuals—including the U.S.—to respond urgently and unequivocally to these attacks, which represent both a serious breach of international legal and humanitarian norms and a threat to public health. In particular, this paper calls for concrete action to:

- *Respond immediately to reduce the risk of chemical weapons being used in future conflicts*: The repeated and increasing use of chemical weapons in the Syrian conflict constitutes a disturbing break with a clear and long-standing prohibition which threatens to set a dangerous precedent for future conflicts. If the use of chemical weapons is allowed to become more commonplace, it will seriously threaten the life and health of civilians, humanitarian responders, and military personnel in conflict settings worldwide.
- *Continue to support international mechanisms for monitoring and enforcing the prohibition on chemical weapons*: Organizations such as the OPCW are critical to monitoring and enforcing international prohibitions on chemical weapons. For this reason, the U.S. and other concerned states should provide robust political, financial, and other support to these institutions, including UN bodies playing a key role in disarmament.
- *Support nonproliferation efforts*: Such efforts include national cooperative threat reduction programs to prevent the proliferation of chemical, biological and other prohibited weapons in and from Syria, and to increase the capacity of neighboring states to interdict weapons transfers.
- *Strengthen commitment to reducing national stockpiles*: States—foremost being Russia and the U.S.—which remain behind schedule for completing full disarmament under the CWC should redouble efforts to reach the target of 100% disarmament. This will send a positive signal to other states that chemical weapons have no place in warfare or military arsenals.
- *Review and support the preparedness of first responders, humanitarian organizations, and military forces*: Given the involvement of an array of military, humanitarian, and other actors in the Syrian conflict, as well as the transnational threat of chemical weapons attacks in the region, it is imperative to review and support preparedness to respond in the event of any future chemical attack. Responders must have up-to-date preparedness training, personal protective equipment, and medical resources, including adequate and readily available supplies of antidotes including atropine and oximes.
- *Support efforts toward criminal accountability for serious violations of international law in Syria*: The U.S. and other concerned states and organizations should play a central role in supporting accountability efforts at both the international and national level for the use of chemical weapons as well as other serious violations of IHL.
- *Support refugees and other victims of the conflict*: The use of chemical weapons in Syria highlights the brutal impact of the conflict on civilians, and the need for states to uphold their commitments to refugee protection in accordance with international law and humanitarian norms, as well as to support other refugee-hosting countries.

## Abbreviations

2-PAM: Pralidoxime
CWC: Chemical weapons convention
ICC: International criminal court
IHL: International humanitarian law
OPCW: Organisation for the prohibition of chemical weapons
R2P: Responsibility to protect
SC: Security council
UN: United Nations
